# Social Listening and its Issues: What can the Precautionary Principle Advice?

**DOI:** 10.1007/s41649-025-00369-x

**Published:** 2025-06-11

**Authors:** Hai Thanh Doan

**Affiliations:** Bioethics Centre, University of Otago, Dunedin, New Zealand

**Keywords:** COVID- 19, Social listening, Precautionary principle, Mass mobilization (Dân vận), Surveillance

## Abstract

The World Health Organization (WHO) has recently initiated “social listening”. The first section of this paper investigates conceptual aspects of social listening. It demonstrates that the WHO’s descriptions of social listening are vague and inconsistent. Notwithstanding this, *possibly*, the WHO-envisaged social listening is constituted by three core components: (i) listening and monitoring, (ii) understanding, and (iii) engaging and nudging. It follows that there is an inherent relatedness between WHO-envisaged social listening and other “social-listening” activities. It follows that to investigate issues of or related to social listening, the inquiry should be broadened to general practices of “social listening”, and experiences related to these must be considered. In the second section, this paper finds several issues with or related to social listening, including bad faith uses, the difficulty of identifying misinformation and punishing it, the echo chambers problem, issues concerning nudging, concerns about policy preset position, concerns for the management and prioritization of resources, and concerns about overlapping between social listening activities. Thus, social listening should be subject to certain rules. In the third section, this paper argues that social listening should be subject to the precautionary principle. Doan, Nie, and Fenton projected that the central teleology, the purpose, and the modus operandi of the precautionary principle could be identified in various policy and legal instruments and propositions, accordingly, the precautionary principle entails, *inter alia*, proactive preparation for public health matters, specifically emergencies, and assessment, e.g. risk–benefit analysis, taking into account uncertainty and past experiences. They showed the normative validity and necessity of applying the precautionary principle in its “*moderate versions*” to public health matters. It follows from this and the rationale underlying and the range of rules of the precautionary principle that the precautionary principle can offer some insights, solutions, and mechanisms to remedy issues posed by or related to social listening.

## Conceptual Aspects of Social Listening

The WHO has recently initiated social listening. The box below provides the titles of the documents and their respective descriptions of social listening:

**Table:** 

Document	Description
Finding the Signal through the Noise: A Landscape Review and Framework to Enhance the Effective Use of Digital Social Listening for Immunisation Demand Generation (Chaney et al. 2021)	“[Social listening is] the regular and systematic aggregation, filtering and monitoring of conversations and public discourse in a combination of traditional media, digital media, off-line and on-line sources of information that represent different populations and geographies” (p.5) [*Emphasis added*] “Social listening is the systematic *monitoring of public discourse and sentiment* as expressed in traditional media, digital media, off-line and on-line sources of information that represent different populations and geographies” (p.7) [*Emphasis added*] “Social listening *is not an end in itself*—it must be embedded in the wider health system so it can respond to the needs and concerns of the population” (p.6)[*Emphasis added*] “The private sector has demonstrated that there is value in advanced digital data gathering approaches for commercial activities, but these are *just recently* being explored for use with public health programmes” (p.6) [*Emphasis added*] “Social listening is used successfully *in commercial marketing* to identify the desires of clients, to design goods and services that fulfil those desires and to develop messaging that generates demand for products … *Social listening can be used to understand how people think and feel about vaccination,* suggest the best ways to *influence public perceptions* and encourage more supportive social processes, all to improve the motivation to be vaccinated” (p.15) [*Emphasis added*] “Often the most important barrier to realising improvements in health behaviours is the gap between knowledge and practice, which in the case of immunisation demand may require interventions that *target practical barriers such as reminders, nudges or incentives that fall outside of the education and communication domain*” (p.19) [*Emphasis added*] “In the centre of this spectrum are the majority of the population who are either accepting of vaccinations or have some reservations and legitimate concerns that make them hesitant to receive or present their child to receive a vaccine. *This group of vaccine hesitant individuals has been called “the movable middle”—those who can likely be nudged* along the curve to become vaccine acceptors” (p.19) [*Emphasis added*] In the conclusion: “Digital social listening is a relatively new technology development which has only recently been used for public health” (p.36) “Social listening technology, especially using Artificial Intelligence, will continue to evolve…” (p.36) “The objective of social listening activities and the infodemic situation in the local context will dictate the focus of communi cations and behavioural interventions, whether to increase trust and encourage the movable middle to accept vaccination or to mitigate and prevent the spread of harmful disinformation and rumours” (p.21)
WHO fourth global infodemic management conference: Advances in social listening for public health, May 2021 (WHO 2023c)	“Social listening is an increasingly important *tool for understanding the ideas and sentiments that people share online*, improving understanding of public opinion so that communication and engagement strategies can better fit people’s needs, addressing their concerns before they are amplified or lead to harmful practices and, and helping undecided or confused citizens to adopt public health and social measures – including vaccination – that protect them.” (p.vi) [*Emphasis added*] “Social listening methods have evolved in recent years, *based on services for corporate communication, marketing, and brand management*. The techniques and tools that have been developed in this context must be adapted if they are to be applied to health, to serve communities and improve health emergency preparedness and response. There is a need for more clarity about the best practices and tools to improve social listening and social network analysis for public health” (p.vi) [*Emphasis added*] “Social listening in Guinea includes an important non digital component” (p.4) “Social listening … It is important to have a have a structured approach to listening” (p.16)
WHO Kicks off Deliberations on Ethical Framework and Tools for Social Listening and Infodemic Management (WHO 2023d)	“Social listening in public health is the process of gathering information about people’s questions, concerns, and circulating narratives and misinformation about health from online and offline data sources”
WHO fourth global infodemic management conference: Advances in social listening for public health, May 2021 (WHO 2023c)	“Developing social listening approaches to understand communities’ ever-changing questions, concerns and narratives can make public health emergency response more adaptive and effective”
An overview of infodemic management during the COVID- 19 pandemic, January 2020–July 2022 (WHO 2023a)	“*Online social listening* requires new open-sourced *tools* that can track and analyse public conversations on social media, blogs and news commentaries, and can disaggregate data by categories such as the complaints people are making or questions people are asking” (4) “*Integrated analysis* of *social listening and other data sources* to generate infodemic management recommendations has rapidly matured during the pandemic” (p.4) [*Emphasis added*]
How to build an infodemic insights report in six steps (WHO and UNICEF 2023)	“Social listening and *infodemic management approaches* can help us understand that noisy information landscape and understand how it’s affecting different populations, and provide evidence-based recommendations for action” (p.43) [*Emphasis added*] “Consider integrating *other social listening reports*” (p.46) [*Emphasis added*]

It can be observed that the instruments of the WHO are inconsistent in various aspects concerning their descriptions of social listening.

*First,* in one WHO’s instrument, the general definition of social listening can be effectively captured as solely listening (Chaney et al. 2021, 5). However, it does not seem that there have been attempts to distinguish conceptually between social listening and listening and related concepts, such as infodemic management, monitoring, or surveillance, and collecting, gathering, and analyzing data. However, also in that instrument, social listening is said to be “*not an end in itself*” but must serve instrumental purposes, i.e. *engaging and nudging* (Chaney et al. 2021, 6, 36). Meanwhile, another definition incorporates the instrumental purpose into the definition of social listening (WHO 2023c, vi). It can be asked whether the instrumental purpose forms part of the concept of social listening or not and whether the definition of social listening as solely listening is accurate or fully captures all conceptual aspects of social listening. Yet, if it is accepted that social listening must serve an instrumental purpose, it is constituted by three core components: (i) listening and monitoring, (ii) understanding, and (iii) engaging and nudging (e.g., (Chaney et al. 2021, 33, 43)). However, if this is so the case, the concept is unclear and awkward:

**Table:** 

[Socal] listening = listening + (engaging and nudging) [presuming that understanding is subsumed into listening]	

This issue arises because of the concern that outcomes of listening must be translated into practical policy actions. That concern is not unreasonable, but if it is so the case, it can be questioned whether social listening is, *conceptually*, the appropriate term.

*Second*, in some documents, social listening is described as “approaches”, while in others, it is described as “a tool”, “a process”, or “something comprising of tools”. Apparently, the phrase “approach” is usually more abstract, broad, and general, while the phrase “process” refers to a specific process and “tool” refers to a specific tool. It is also unclear whether the inconsistent use of plurality and singularity has any implications. It is not meant to suggest that social listening cannot be referred to by all of these terms, especially in different contexts, but more clarity should be demanded.

*Third,* social listening is sometimes described as comprising both online and offline activities, both digital and non-digital tools. Yet, in some documents, the description is that “Social listening is … *tool for understanding the ideas and sentiments that people share online*” (WHO 2023c, vi) [*Emphasis added*].

*Fourth*, in a document, while it is stated that social listening is something novel in public health which is developed from commercial private activities (WHO 2023c, vi), it is acknowledged that the unstructured gathering of data may have happened for a while and “*digital social listening is a relatively new technology development* which has only recently been used for public health” (Chaney et al. 2021, 36). Though some documents suggest that social listening also entails unstructured gathering of data, some others articulate that social listening aims to “have a *structured approach* to listening…” (WHO 2023c, 16). A significant portion of the document “*Finding the Signal through the Noise A Landscape Review and Framework to Enhance the Effective Use of Digital Social Listening for Immunisation Demand Generation*”, as the title suggests, is dedicated to digital approaches to social listening, and the historical account of social listening is also premised on digitality (Chaney et al. 2021, 6, 15). Considering these matters, it is unclear whether the WHO prepares instruments to envisage and guide “social listening” or “digital social listening” and even whether social listening referred to by the WHO is only “digital social listening” or also comprises “non-digital social listening”.

Meanwhile, the historical account of social listening sketched by the WHO and from which the WHO envisages its social listening does matter the substance of WHO’s social listening and its potential ethical and legal issues. In marketing scholarships, it is a general consensus that social listening [in commercial private activities] refers to tools related to social media, digital technologies, and platforms (Mỹ 2022; Kompa 2023; Reputa 2020; Brand24 n.d., chap. 1; Kim 2020; Kotler 2017, 110–11). Although social listening in commercial practices relates to commercial purposes and changes in the precise operation of the brands or businesses, it has no intrinsic connection with nudging. For example, if a product or brand is working well, there seem to be no compelling reasons for [more] nudging or changes, which costs resources, or if a product or brand is not working well, sometimes, the solution is the cessation or dissolution of a product or brand. Meanwhile, social listening in private practices does not relate to public coercive powers and public resources similar to any activities in the public sphere like social listening envisaged by the WHO. Private commercial social listening is conducted out of the pocket of private entities, and for this reason, private entities have strong reasons to limit social listening in balance with the purpose for which social listening takes place and the entities’ resources.

Meanwhile, if social listening is constituted by three core components (i) listening and monitoring, (ii) understanding, and (iii) engaging and nudging, regardless of the precise mediums, it can be asked whether the WHO’s social listening can find alternative historical accounts. It can be argued that there are other social-listening practices that share more similarities and even are identical to the WHO’s social listening compared to social listening in private commercial practices, and for this reason, it is more accurate for the WHO’s social listening to be developed from or to find counterparts in these practices. In one of the bible works in comparative law written by Konrad Zweigert and Kötz in 1960, functionalism – one basic and fundamental approach and method in comparative law – premises on three observations and theoretical presumptions: “(i) the system of every society faces essentially the same problems, and (ii) [they] solves these problems by quite different means though very often (iii) with similar results” (Zweigert et al. 1998, 3:34). Social surveillance and monitoring, mass mobilization (also known as social mobilization), or “dân vận” in Vietnam (Hoài 2021; Minh 1949, 1953) or 社会动员机制 in China (Tsai 1999) are practices found across countries and regions which, though having different names, are similar or identical in nature, which is shared by WHO’s social listening. It follows that the ambiguity of the WHO-envisaged social listening, and the inconsistencies between descriptions of social listening can be assisted by referencing related other social-listening practices.

Vietnam’s practice of “dân vận” shall be used to demonstrate the points. Dân vận (mass mobilization) comprises listening plus encouraging or nudging. In the article entitled “Dân vận”, it was coined that “*Mass mobilization is mobilizing all the forces of each citizen without leaving a single citizen out to contribute to the whole people's force, to perform the work that should be done, the work assigned by the Government and the Union*” (Minh 1949) [*Emphasis added*]. “*Mass mobilization* is, first of all, to *find ways to explain* clearly to each person that each and every work is for the people’s benefit and the work is people’s duty so that they must enthusiastically do it. Secondly, *all government officials, all union officials and all members of people’s organizations must monitor, help, urge and encourage people*” (Minh 1949) [*Emphasis added*]. Social listening, monitoring, and being aware of public opinion and sentiments are crucial for mass mobilization (Mạnh 2023). In light of the vision, propaganda committees and commissions for mass mobilization have been found whose mandates are, inter alia, listening and firmly grasping public opinions and fighting against distorted voices (Khánh 2024). The Vietnamese Communist Party and the State also fund the so-called Force 47 and contract private public-relation services for similar purposes (Hoa 2017; Hookway 2017; Reddit 2024). Vietnam has also cooperated with Facebook to remove or limit posts it considers negative, including dissident voices, in a manner similar to the description of WHO’s social listening that “social listening is “supported in a partnership with Facebook Data”” (Chaney et al. 2021, 29; BBC News Vietnamese 2021a). Vietnam also utilized and termed accurately “social media listening software” [phần mềm lắng nghe mạng xã hội], distinguished from related concepts such as “dân vận”. According to the Government, “the software shall collect data from a series of popular social networking platforms” (*Thanh *Niên 2024) and “is also integrated with artificial intelligence (AI) and machine learning” (*Thanh *Niên 2024). “The software is capable of identifying the opinions, thoughts, moods and emotions of the community with positive, neutral and negative nuances, as well as trends on the network” (*Thanh *Niên 2024). “The software can help shape and ‘manage’ public opinion” (*Thanh *Niên 2024), “especially the content and information developments of hostile subjects” (*Thanh *Niên 2024). That is, the novel digital tool assists the age-old practices. Thus, it seems that Vietnam makes clearer conceptual distinctions between “dân vận” and other concepts such as listening, encouraging and nudging, and social media listening.

Meanwhile, the WHO also intentionally narrows down social listening for it to serve certain mentioned public health objectives, i.e. social listening related to public health programmes: “the techniques and tools that have been developed in this context must be adapted if they are to be applied to health, to serve communities and improve health emergency preparedness and response” (WHO 2023c, vi). However, on the one hand, it is not clear whether the WHO intends to develop its own social listening or that “for public health purpose/programmes” is a constituent of the concept of social listening; currently, one definition incorporates it while the others do not. Meanwhile, it is clear that the list of mentioned purposes, i.e. handling infodemic and promoting vaccination uptake, is not exhaustive (e.g. behaviour changes concerning climate change, sexual and reproductive health, gender equality, and mental health are also mentioned (Chaney et al. 2021, 11)), but it is not clear as to which “for-public-health” purposes or activities are excluded from social listening. Inasmuch as the WHO intends to develop its own [public health] social listening, the scope of social listening can be a question. On the other hand, whether the “quantitative changes lead to qualitative changes”, i.e. the changes in relation to techniques and tools and purpose, yield a new variant of [public health] social listening separate from other social-listening practices or whether the WHO can really invent a separate and separated “public health” social listening, is not clear. Notwithstanding WHO’s view, social listening, without proper safeguards, can be used by various actors, not exclusively health authorities, and for various purposes, not exclusively public health.

Meanwhile, inasmuch as the historical account of social listening as sketched by the WHO overlooks the broad practices of “social listening”, ethical and legal issues related to the WHO-envisaged social listening are not sufficiently unpacked or taken seriously. It can be argued that unethical issues related to social listening should not be considered as social listening recognized by the WHO. However, the WHO is not de jure competent or de facto capable of controlling the use of social listening, nor can it be held accountable for any use of social listening. Consequently, aside from WHO’s view, there should be principles and mechanisms in place to guarantee the validity and legitimacy of social listening. It follows that to investigate issues of or related to social listening, the inquiry should be broadened to general practices of “social listening”, and experiences related to these must be considered.

## Potential Issues of, or related to Social Listening

It should be made clear in advance that what is discussed in this section are “potential issues” which might or might not occur, depending on specific acts of the policy-makers and enforcers and relevant checks. The potential issues are potentially posed by or relevant to social listening, although they are not exclusively due to or restricted to social listening.

### Bad Faith Uses

As a tool, social listening is not immunized from malicious/bad faith uses*.* Singh (2024) demonstrated a case of bad faith use in private activities. Concerns that governments maliciously use social listening have been persisting and have never ceased, e.g. as satirized in the fiction “1984” by George Orwell: “*Big Brother is watching you*”. Indeed, the WHO’s reports acknowledged potential abuse of social listening, such as surveillance, identification of citizens with “deviant” opinions, and predictive modelling as a first step towards controlling and censorship (WHO 2023c, 19–20). Indeed, without appropriate mechanisms, tools envisaged to be used by public health authorities can be misused, e.g. social listening is used to identify critical voices, then it directs social norms to extinguish the critical voices (see the “The Slippery Slope of Nudging” section).

### Signals and Noises: Misinformation vs Information?

In theory, attempts to counter disinformation are not quite problematic since the malicious intention distinguishes it out (but in practice, it can be trickier than in theory). Notwithstanding, attempts to counter misinformation can be challenging. Misinformation, per definition, is false information shared by people who do not realize it is false and do not intend to cause any harm (Wardle and Derakhshan 2017). Meanwhile, human beings are still far from comprehending the universe. That means there is a—at least theoretically speaking—possibility that scientific information is falsified. In fact, science progresses precisely because previously widely believed scientific truths are falsified. Nevertheless, it is doubted that the has-been-proven false science should not be circulated, trusted, and can even be sanctioned, when it was first coined for the reason that, theoretically, it will be falsified later. The “butterfly-wing effect” casts greater doubt upon this. That raises the question of when a piece of information should be labelled as misinformation, countered, and gotten rid of, especially in the insufficiency of information and uncertainty. That also raises the question of how early signals can be attended to and what are precise responses to early signals made in the insufficiency of information and uncertainty.

It is helpful to recall experiences concerning COVID- 19 in this regard. Around November 2019, Dr Li Wenliang sent a piece of news on a private WeChat group to about 100 medical staff, his ex-classmates at the university, which read: “*7 cases of SARS have been confirmed*” (Nie and Elliott 2020). The message spread across Weibo. Dr Li faced reprimand, was detained by police, and shamed on national television (Nie and Elliott 2020). Though, undoubtedly, Dr Li and several other doctors who had tried to ring the alarm are heroes; it is hard to say that Dr Li’s information is accurate, supposing that we temporarily put aside the hindsight knowledge about the devastating impacts of COVID- 19 and certain negative impressions we may have against the Chinese Government, but instead using a critical investigation based on a strong distinction between accurate fact an inaccurate fact (misinformation). Undoubtedly, Dr Li would have had been right had he stated that “a disease has emerged”, but the message “7 cases of SARS have been confirmed” was not accurate. Is it justified for Dr Li to be censored, punished, made a case, and educated, given that he communicated misinformation? Likewise, the public statements of Chinese authorities that the new disease (which was later named “SARS-COV- 2”) was not SARS but a new coronavirus which could not transmit readily between people [and could not cause severe illness for many] (WHO 2020; Belluz 2020) were hardly to be asserted as wrong. They got it right that SARS-COV- 2 is not SARS. Although, with hindsight knowledge, Chinese authorities did not get it right in claiming that “a new coronavirus which could not transmit readily”; notwithstanding, strikingly, at the time before more knowledge concerning SARS-COV- 2 came to light, the general knowledge was that coronavirus is a type of common virus that typically causes only mild to moderate upper respiratory illness, with only exceptions are SARS and MERS (UN High-level Panel on the Global Response to Health Crises 2016). It can yield sensational opposition in this regard, but it is not wrong to assert that the statements of Chinese authorities were not erroneous in light of the available knowledge at that time (though this can be countered should there be evidence that Chinese authorities had more knowledge than the general and common knowledge at that time about the new virus). The distinction of information-misinformation (accurate-inaccurate) information cannot do justice to the case of Dr Li and similar cases. If there are any doubts, just imagine a counterfactual history where the disease caused by the new pathogen could be eliminated in some days and was not disastrous. Would the removal of the information provided by Dr Li and his punishment be more justified as a matter of principle?

However, if we keenly follow the line of logic, we can see that misinformation is more likely than accurate information, and it would also follow that it is hard for citizens not to be sanctioned in circulating information where the information is insufficient and uncertain. In many instances, there is no clear and meticulous signal. Thus, if organizations and states refuse to listen to jam signals, there is a possibility that they will turn deaf ears to all signals. And, if social listening is used to counter and prevent misinformation by furnishing itself the power of sanction (even if the sanction is not implemented), it might curb the flow of information and even prevent proper and due assessment and preparation for risks and dangers as well as prevent the genesis of new knowledge which is often the accumulation of falsified information.

### Echo Chambers

Considering that social listening comprises nudging people, removing misinformation, and, within the Vietnamese context, shaping and managing public opinion, there are significant risks of echo chambers. That is, the authorities shall only listen to the voices they shape or nudge or its own voice or only the voices they want to hear. This matter is more significant in relation to digital approaches. Social media is not generally accessible, especially to the worst-off people (the problem of *inequitable access*), and since it is not generally accessible, it is not representative. This amplifies and concretizes mainstream voices at the expense of unheard and marginalized voices (WHO 2023c, 7).

### The Slippery Slope of Nudging

According to Sunstein, nudging is an easy and not costly way to influence and alter people’s behaviour into a perceived desired or good behaviour without “forbidding any options” or formal punishments (Thaler and Sunstein 2008, 6). Sunstein himself seems very confident in nudging, seeing it as “*liberty-preserving approaches*” (Sunstein 2014, 583). He drew considerations for nudging, namely, transparency and effectiveness, based on evidence and testing. Sunstein’s view is not without doubts: one instrument of the WHO concerning social listening—though it does not assert—gently urges “consideration of the role of censorship and the various conflicts between providing information, nudging people towards the desired behaviours, “blaming and shaming,” etc.” (WHO 2021, 19, 20) [*Sic*]. In fact, contrary to Sunstein’s view, nudging is not inherently ethical and is not without repercussions. The absence of formal punishments cannot be equated to immunity from coercion and punishment, particularly psychological ones: *nudging can be coercive and harassive*. The specific purposes and methods of nudging determine or influence its ethicality and legitimacy. Many real-world examples and aspects can be epitomized and analyzed, but for the sake of conciseness, the Việt Á Grand Corruption Case shall be used to demonstrate the point (another issue is briefly mentioned in the section concerning “Policy Preset Position”). Early 2020, public authorities wrongly announced that Việt Á’s KIT was approved by the WHO and the UK Department of Health and Social Care for circulation in European Countries (Thúy Hà 2020; Thảo Lê 2020; LT 2020). Actually, this information could be checked online (there are online portals and notifications of review outcomes from WHO and the UK Department of Health and Social Care). In fact, some people were well aware of the fact that the KIT was not approved by the WHO (BBC News Vietnamese 2021b). Nevertheless, attempts to unpack the matter were subjected to a chilling environment: when someone cast doubt on or attempted to expose the matter, they would be lapidated by or reported (to Facebook) by a mass of people. Only nearly two years later, the true information came to the attention of the public as a byproduct of charges brought against bribery activities. There were no formal sanctions imposed by the Party-State on criticisms against the KIT; traces of police power could hardly be found, nor were there straightforward violations of rights, which are situations that Sunstein conceded that nudging attempts should be reconsidered (Sunstein 1996). The case clearly relates to the WHO-envisaged social listening inasmuch as (i) the State conducted dân vận (mass mobilization) including social listening and social listening software and nudging—Sunstein himself described “uses of social norms” as among nudges (Sunstein 2014, 586); (ii) concerns against the KITs can be labelled as misinformation by the mass; and (iii) nudging is not without repercussions: it can create social norms against frankness and free flow of information as its byproduct. Obviously, if “KITs” in the case is substituted by “a specific newly-developed vaccine”—and not vaccines in general—the relevancy of the mentioned case to the WHO’s social listening is transparent. There is no reason to think that different to KITs, the production of vaccines cannot be subject to sub-standard, misconduct of manufacturers, and corrupted behaviours of certain authorities.

### Policy Preset Position

Social listening recognized that there are “legitimate concerns that make them [*people*] hesitant to receive or present their child to receive a vaccine” (Chaney et al. 2021, 20) [*connotation added*] and “systematic social listening activities to understand and monitor public sentiment about vaccines can be very useful for understanding the specific concerns of the movable middle. They may be receiving misinformation or have concerns about safety and efficacy influenced by local and global dialogue” (Chaney et al. 2021, 21). However, unfortunately, the report goes on to focus only on nudging these people. It seems to consider these people as have been exposed to “harmful misinformation, disinformation and hoaxes” (Chaney et al. 2021, 21) and for this reason, action is taken “to correct, contain and minimise the spread and negative effects of harmful misinformation, disinformation and hoaxes” (Chaney et al. 2021, 21). This gives the impression that such people are subjects for mere nudging. It fails or at least does not make clear unequivocally that these legitimate concerns can suggest real issues which are under-explored or need to be reconsidered by the relevant authorities. This gives an impression of a presupposition that authorities are superior to the citizenry and shall nudge the citizenry towards desired behaviours and accurate information, which can sometimes be problematic (see examples for this in Doan 2023). More problematic, before, during, and after the course of listening, there is a presupposition that people who are listened to, to the extent of not being aligned with the available knowledge of experts, have been subjected to misinformation. Hardly can this be a proper presupposition or attitude. This attitude can even incline to preset or set a policy position rigidly.

The problem of policy preset position is real and has taken place. For example, in the late middle of the COVID- 19 pandemic, Vietnamese National Television (VTV), in response to criticisms to reconsider restrictive measures, claimed that “*Living with COVID- 19 is the rhetoric of the rebels*”. Vietnamese authorities did listen to the society (and knew that there were people opposing the policy approach on certain grounds), but it asserted that such people were rebels or at least were influenced by misinformation and disinformation [of the rebels] and chose to counter these positions. This case also illustrates the ethical and practical problems with the policy preset position and the twin purpose of listening and nudging. If there is a preferred policy preset or a predetermined position and social listening is for the sole purpose of nudging people to accept that position or change their mind and implementing the policy preset, social listening is a mere surveillant tool for oppressiveness.

However, if policy-makers turn deaf ears or listen with a too strong and rigid subjective view or preset their policy position/approach firmly and with thoughts that other people are influenced by misinformation or disinformation, then they might well act arbitrarily, considering existing uncertainties and the constant changes of the context.

### Management and Prioritization of Resources

All activities spend resources. Issues concerning the management and spending of resources are self-evident for any activities. Also, it is self-evident that if resources are overused and wasted, repercussions inevitably happen. Though not often costs and expenses are, stand-alone, a reason for rejecting the activities or technologies, the appropriate use of resources for activities or technologies is always a concern and always needs to be reminded. Considering the WHO’s vision that social listening can be a regular or even daily activity and combine various digital and non-digital activities (Chaney et al. 2021, 5, 11), this potential risk is not negligible or insignificant. Although attempts to collect data to understand the public’s sentiment and concerns are sensible, there are questions as to the justified extent of resources dedicated to these attempts, particularly when these attempts should cease to be justifiable.

Meanwhile, considering the special attention the WHO pays to digital social listening, it can also be asked how much resources should be paid to digitality, especially by the least worst-off countries or localities which are still struggling with starvation and devastated medical facilities.

### Overlapping

Considering the thick categories of social listening and social-listening-related activities and the distinction between public health social listening and other social listening practices, the risks of overlapping and wasting resources are not insignificant.

In light of various ethical and practical issues, social listening should be subject to certain rules.

## Implications of the Precautionary Principle

### The Precautionary Principle

It is commonly believed that the official history of the precautionary principle started with West German environmental law’s articulation of “Vorsorgeprinzip” (or “foresight principle”) (Bourguignon 2015; Martuzzi 2007; Boehmer-Christiansen 1994; von Moltke 1988).^1^ “Sorge” means “worry”, “concern”, or “care”. Sonja Boehmer-Christiansen (1994, 38) explained diligently and carefully why care for the future leads to preparing for the future, but the care alone does not sufficiently capture “Vorsorgeprinzip” but acts of anticipating and preparing for the future (“foresight”).

The precautionary principle is considered by some people as has been defined differently by different instruments or documents. Some well-known statements are as follows:

Principle 15 of the Rio Declaration of the UN Conference on Environment and Development reads:

In order to protect the environment, the precautionary approach shall be widely applied by States according to their capabilities. Where there are threats of serious or irreversible damage, lack of full scientific certainty shall be not used as a reason for postponing cost-effective measures to prevent environmental degradation.

Article 3.(3) of the UN Framework Convention on Climate Change (UNFCCC) reads.

The Parties should take precautionary measures to anticipate, prevent or minimize the causes of climate change and mitigate its adverse effects. Where there are threats of serious or irreversible damage, *lack of full scientific certainty should not be used as a reason for postponing such measures*, taking into account that policies and measures to deal with climate change should be *cost-effective* so as to ensure global benefits at the lowest possible cost. To achieve this, such policies and measures should *take into account different socio-economic contexts, be comprehensive,* cover all relevant sources, sinks and reservoirs of greenhouse gases and adaptation, and comprise all economic sectors. Efforts to address climate change may be *carried out cooperatively by interested Parties*. [Emphasis added]

The Wingspread Statement reads:

When an activity raises threats of harm to human health or the environment, precautionary measures should be taken even if some cause and effect relationships are not fully established scientifically. In this context, the proponent of an activity, rather than the public, should bear the burden of proof. The process of applying the Precautionary Principle must be open, informed and democratic and must include potentially affected parties. It must also involve an examination of the full range of alternatives, including no action”. (Ashford et al. 1998)

In a recent paper, Doan, Nie, and Fenton, in investigating legal-policy instruments and relevant practices and behaviours concerning infectious diseases, public health emergencies, and COVID- 19, demonstrated that the central teleology, the purpose, and the modus operandi of the precautionary principle could be identified; accordingly, the precautionary principle entails, *inter alia*, proactive preparation for public health matters, specifically emergencies, and assessment, e.g., risk–benefit analysis, taking into account uncertainty and past experiences (Doan et al. 2023). They argued for the normative validity and necessity of applying “*the moderate versions*” of the precautionary principle to public health matters while opposing what they considered “*the hard versions*” (Doan et al. 2023).

Doan, Nie, and Fenton’s “the hard versions” are qualitatively different from “the strong versions” of Sunstein and many other scholars (Sunstein 2003, 1012–13; Hughes 2006). These scholars believed that the Wingspread Statement is “a strong version” (Sunstein 2003, 1012–13; Hughes 2006). However, it should be recalled that statements of general principles are abstract and should not be intolerable because of abstractness. Obviously, statements of general principles sometimes cannot capture all aspects of the principles, nor can they be formulated in such a detail similar to specific legal provisions. Notwithstanding, from a legal perspective, it is strange to think that without guidelines being mentioned in its definition, the precautionary principle, which is an abstract idea, shall be implemented alone within the landscape of a vacuum of rules or shall override all other considerations, legal principles, and legal tests, thus acting on extremes (acting on extremes seems to be one of Sunstein’s arguments against the precautionary principle). The precautionary principle must always be read within a legal landscape and factual context and together with certain considerations, legal principles, and legal tests regardless of whether the latter are considered internal (i.e., components for constituting the principle) or external (i.e., those supplementing, delineating, or refining the principle). Notwithstanding that, the Wingspread Statement itself is not unreasonable if it is read in and with consideration of factual contexts. There can be broad categories of scientific uncertainties and insufficiency of information, not exclusively “[s]ome cause-and-effect relationships are not fully established scientifically”. The phrase makes sense in contexts such as ozone layer depletion or COVID- 19, which are not limited. The arguments that measures, including research to clarify the issues, which obviously cost resources, shall not be implemented until there is full scientific certainty are hardly reasonable (and *if a matter is of complete and unchallengeable scientific certainty, what is the purpose of studying the matter?*). Similarly, “the proponent of an activity, rather than the public, should bear the burden of proof” is not unreasonable. It is not unreasonable for proponents of an activity—which impacts fundamental interests and rights of various subjects to various extents—who control the data of safety, e.g. for human use and for the environment, to provide the data. It should be noted that the Wingspread Statement only encourages or even requires measures but says nothing about the nature of the measures in such a manner that can threaten to contradict other rules and principles (inasmuch as the latter are considered external to the precautionary principle).^2^ Doan, Nie, and Fenton’s “the hard versions” refer to something *truly unreasonable and unacceptable* which are due to, for example, the recklessness or wilfulness of “*instrumental mentality*”, willing to implement certain propositions at all costs, regardless of sufferings (Doan et al. 2023).

Doan, Nie, and Fenton’s line of normative analysis echoes the official positions of the European Union (EU) and various jurisdictions. To guard against undesirable use of the precautionary principle (“the hard versions” in the words of Doan, Nie, and Fenton), the European Union and other jurisdictions subject this principle to guidelines of which the satisfaction indicates the proper application of the precautionary principle (in Doan, Nie, and Fenton’s word, “the moderate versions”). Meanwhile, the UNFCCC considers such “guidelines” as components constituting the precautionary principle. These guidelines and components help consolidate, progressively develop, and refine the precautionary principle and help it overcome the “*inherent ambiguity of language*” (Hart 1961, 124).

#### Risk assessment

According to the European Court of Justice (CJEU), precautionary measures cannot properly be based on a purely hypothetical approach (CJEU 2002, paras. 143–145). There must be a risk assessment (CJEU 2002, para. 148). The competent public authority must ensure that any measures that it takes are based on as thorough a scientific risk assessment as possible (CJEU 2002, para. 162). Scientific advice must be based on the principles of excellence, independence and transparency (CJEU 2002, para. 159). According to WTO Appellate Body, “Risk that is to be evaluated is not only risk ascertainable in a science laboratory operating under strictly controlled conditions but also risk in human societies as they actually exist, in other words, the actual potential for adverse effects on human health in the real world where people live and work and die”(WTO Appellate Body 1998, para. 187). According to CJEU, to the extent to which an authority opts to disregard a scientific opinion, an authority can “base its decision on a supplementary opinion from the same experts or other evidence, whose probative value is at least commensurate with that of the opinion concerned” (CJEU 2002, para. 159).

#### Provisional Measures in Uncertainty

The precautionary principle is inherently linked to provisional measures in uncertainty. According to CJEU, “[w]here there is uncertainty as to the existence or extent of risks to human health, the institutions may take protective measures without having to wait until the reality and seriousness of those risks become fully apparent”(CJEU 1998, para. 99). Similar opinions and holdings can be found in different jurisdictions; for example, in the OSPAR Arbitration Case MOX Plant, it is opined that “Government must apply caution, and take preventive measures even where there is no conclusive evidence of a causal relationship between the inputs and the effects” (Permanent Court of Arbitration 2003, para. 84).

Precautions, however, are needed to differentiate between issues that have been thoroughly addressed and concerns are hypothetical or about theoretical risks vs novel risks in which there is uncertainty and insufficiency of information and issues whose concerns suggest that more information has come to light casting doubt on “whether the previously existing body of scientific evidence still permits of a sufficiently objective assessment of risk” (WTO Appellate Body 2008, para. 703).

Insofar as measures are provisional, they must be time-bounded and not be imposed to be permanent. Thus, provisional measures are subject to review within a reasonable period. Meanwhile, additional information necessary for the purpose of a more thorough assessment of risk must be sought (WTO Appellate Body 2008, para. 676; CJEU 1998, para. 101).

#### Proportionality, the use of Models, Data, and Analysis Methods, and Taking into Account of Social-Economic Factors

Protective measures proportional to the desired level of protection should demonstrate that “benefits outweigh potential damage”.^3^ Meanwhile, [quantitative] cost–benefit analysis is not the only method of assessment or instruments (ILGRA 2002, 16; Doan 2023, 19).

Meanwhile, though it cannot be collapsed into the component of proportionality, the component of taking into account social-economic factors is entailed by the precautionary principle (Article 3.(3) of the FCCC), which, to an extent, relates to, supplements, and bolsters the idea of proportionality. According to the UK Interdepartmental Liaison Group on Risk Assessment (ILGRA), “the examination must take account of social and environmental costs and of the public acceptability of the different options possible”(ILGRA 1998, para. 22). “Serious considerations of representations of impacted people” is a factor to be considered in judging decisions made by public authorities (High Court 2000, para. 56). In certain cases, the authorities are “entitled to take into account public opinion” (Court of Appeal (Civil Division) 2020).

These guidelines and components of the precautionary principle are not baseless or arbitrarily set. They ground on (i) theoretical presumptions of the precautionary principle, namely, (1) in many circumstances, full [scientific] certainty can hardly be attained and information necessary for making decisions is said to be insufficient (when information is said to be sufficient is not free from controversial), (2) human beings are fallible, and (3) situations may change from time to time, and (ii) legitimate expectations of “*responsible, representative governments*” (WTO Appellate Body 1998, para. 124) who act with “prudence” (WTO Appellate Body 1998, para. 124) and “sincerity”.^4^ It follows that the precautionary principle’s ethos is erring on the safe side, and “the safe side” is not static or is arbitrarily set but is determined upon rational considerations; for otherwise, it is not “precautionary” and cannot be justified (See the argument against hard versions in Doan et al. (2023). In light of the theoretical foundations of the precautionary principle, ensuring procedural justice is essential, which is quite similar to any other legal matters (Hart 1961, chap. 8).^5^ Reading guidelines and components of the precautionary principle, it can be observed that substantive justice is also required.^6^

Notwithstanding the fact that certain scholars still contest the precautionary principle strongly,^7^ the precautionary principle is now accepted as *a general legal principle* in certain jurisdictions, notably international law and EU law, whose scope of applicability covers environmental and health issues (European Commission 2000, 2).

### A Contested Connection between the Precautionary Principle and Social Listening?

Any statements as to the connection between [WHO-envisaged] social listening and the precautionary principle can be very controversial. An obvious reason is that WHO’s documents concerning social listening do not mention the precautionary principle. Another reason is that the WHO is also always inconsistent in terms of the precautionary principle. For example, the World Health Report 2002 recognized the precautionary principle as a risk management tool (WHO 2002, 39, 150) and the Fourth Ministerial Conference on Environment and Health held in Budapest, Hungary, from 23 to 25 June 2004, reaffirmed “the importance of the precautionary principle as a risk management tool” (WHO 2004, 6); however, the International Health Regulations Review Committee regarding amendments to International Health Regulations (IHR Amendments RC) indicated that the precautionary principle is contrary to the evidence-based approach and is incompatible with the IHR “Measures in the Regulations are meant to be *evidence-based, which may preclude or at least limit the application of precaution*…” (WHO 2023b, 28, 29) [*Emphasis added*], though it also oddly and contradictorily added that “*uncertainties during an outbreak response may require action in the absence of evidence or with insufficient evidence*” (WHO 2023b, 28, 29) [*Emphasis added*].

Notwithstanding, considering the status of the precautionary principle as a general principle of law whose scope of applicability entails public health, regardless of the precise intention of the WHO as to these matters, the precautionary principle rules practices such as social listening. In *Legality of the Threat or Use by a State of Nuclear Weapons, Advisory Opinion of 8 July 1996*, the International Court of Justice opined that “To ascribe to the WHO the competence to address the legality […] could not be deemed a necessary implication of the Constitution…”(ICJ 1996, para. 25). It can be read from this opinion that though the WHO’s views concerning the precautionary principle and social listening, however inconsistent and contradictory, shall be given regard to as relevant factual factors—*but only as factual factors*—the WHO is not competent to determine the legality of the precautionary principle and social listening. For a general legal principle like the precautionary principle, they do not need to be explicitly articulated to be claimed as being presented in or relevant to tools, documents, or jurisdictions (WTO Appellate Body 1998, para. 124). Moreover, considering guidelines and components of the precautionary principle and conceptual aspects of social listening, it is not unreasonable to observe that what is termed “social listening” by the WHO is what can be done by the invocation of the precautionary principle (although the precautionary principle can indicate more measures). Meanwhile, it is not hard to spot the precautionary thinking in the objective that enshrines “social listening”: Social listening aims to “have a structured approach to listening, *so analysis leads quickly to insight then action before problems become too large and well-established*” (WHO 2023c, 16) [Emphasis added], to address concerns “*before they are amplified or lead to harmful practices*” (WHO 2023c, vi), to “*improve health emergency preparedness and response*” (WHO 2023c, vi).

### Implications of the Precautionary Principle for Social Listening

The precautionary principle has inputs for social listening in aspects including the following:“Measures must *take into account different socio-economic contexts*” entails that efforts should be made to ensure that input to policy is collected on an equitable and inclusive basis.

Similarly, good faith, openness, reflection, and caution should always be appreciated. If social listening is only about listening with the presupposition that those who are listened to have been subjected to misinformation and disinformation and shall ultimately be nudged, no real, critical, and reflexive listening can take place. Consequently, opportunities to learn from the community’s knowledge or for prompt assessment can be reduced. Precautions are also needed concerning the extent of nudging so as not to reduce listening to be mere symbolistic, which does not enable real listening.

Considering the difficulty in drawing the line between information and disinformation, precautions should be needed before ruling out that a piece of information is misinformation. Instead of a binary distinction, a more nuanced and informative labelling system that clearly and detailed states the status of the knowledge relevant to the matters, including grey matters which need further studies and clarifications, should be more desirable.

Meanwhile, it should be acknowledged that fearmongering, overly anxious, and disproportional measures, which are harmful, can be formed upon accurate information. Reactions formed upon public opinions, thus, should be rational and proportionate: it cannot be afforded that concerns for theoretical or insignificant risks can outright ban or suspend certain products, technologies, or measures (WTO Appellate Body 1998, para. 124). Meanwhile, proper communications as to the state of knowledge, especially potential risks, concerning the products, technologies, or measures and proper preparations for anticipatable worst scenarios should be necessary. For example, though we cannot immediately ascertain whether certain concerns about potential adverse effects of certain specific vaccines are valid (and even if accepting that some concerns are valid and undesirable side effects can happen for an unknown probability), inasmuch as the analysis of costs-benefits, and disadvantages-advantages favour deploying such technologies or products, they should be administered or used. Proper communication should be ensured as to what has been known and established about the side effects of vaccines and how likely and unlikely theories concerning certain matters of concern are. Preparations for side effects, such as post-vaccination monitoring and proper and sufficient remedy for side effects, e.g. prompt medical assistance and even compensation, attest and accredit the legitimacy and righteousness of the measures. If it is accepted or presumed that the “movable middle” people, as labelled by the WHO, are reasonable, they shall be convinced by the proper measures backed by sufficient guarantees. And if it is argued that these measures are nudges, the nudges should be proportional, considering that, as argued, there is no reason to think that nudging cannot be aggressive or is self-proportional.

Managing material and human resources is an aspect that needs to be precautionary. Clearly, it is not reasonable for disproportional and excessive resources to be poured into social listening. Nor is it reasonable for resources to be poured primarily into social listening at junctures when other concerns, e.g. improving the welfare of medical staff, patients, and facilities, should be prioritized. Specific social listening activities should also be suspended, and resources should be rechannelled where information is gathered sufficiently for informed and rational decisions to be made. That is also to prevent situations where redundant information can constitutively confuse decision-makers and enforcers with the infodemic—“an overabundance of information from online and offline sources that accompanies an epidemic or other health crisis” (WHO 2021, 2–3)—from occurring. Precautions should be paid to social listening activities in the least worst-off countries or localities which are still struggling with starvation and devastated medical facilities so that social listening shall not pose greater strains on the resources.

To avoid overlapping and wastefulness of resources, cooperation and integration of various social listening activities where appropriate, as opposed to the strict departmental view, should be necessary. Meanwhile, precautions should be taken to ensure properness in the transfer and management of data across departments and entities.

Social listening, or any measures taken amidst uncertainty or to address uncertainty, should still be reviewable. Jurisprudences across jurisdictions, e.g. EU law, provide for grounds that actions can be challenged, such as where such actions are grossly wrong or “manifest error” (CJEU 2002, para. 166) or constitute “a misuse of power” (CJEU 2002, para. 166). Actions can be challenged if the authorities clearly exceed the bounds of their discretion in conducting such actions (CJEU 2002, para. 166), or if the government’s actions lack a reasonable relationship with the evidence upon which they rely, or if governments’ actions are overly disproportionate or arbitrary. Actions can also be challenged where new and extant evidence cast doubt on governments’ actions (CJEU 2007). Consequently, mismanagement of risks and improper measures, including improper nudging, should still be held accountable. It follows that malicious and dubious acts can never be justified and should never be resorted to in any circumstances.

## Conclusion

While finding various inconsistencies and ambiguities as to WHO’s descriptions of social listening, this paper suggests an inherent relatedness between WHO-envisaged social listening and other social listening activities. This paper finds several issues with or related to social listening, including bad faith uses, the difficulty of identifying misinformation and punishing it, the echo chamber problem, issues concerning nudging, concerns about policy preset position, concerns for the management and prioritization of resources, and concerns about overlapping between social listening activities. This paper argues that social listening should be subject to the precautionary principle. Meanwhile, the precautionary principle can offer some insights, solutions, and mechanisms to remedy issues posed by or related to social listening.

## Data Availability

Not applicable.

## Footnotes

1 Actually, the precautionary principle can be argued to have a longer history which this paper shall not address in detail (Haigh 1994).

2 One of the implications of the foregoing analysis is that, for example, depending on the precise potential risks (and also the countervailing risks), and the completeness of data prepared and provided by the proponent of the activity, various administrative propositions can be made concerning an activity or product, ranging from banning, conditional use, conditional authorization, emergency use, emergency authorization, unconditional use, and authorization as opposed to the single solution of banning.

3 It should be noted that the proportionality principle and the precautionary principle are two separate concepts and are conceptually distinguished. Yet, there is an overlapping between the two concepts. According to the European Commission, proportionality is one of the general principles of application of the precautionary principle (European Commission 2000, sec. 6.3; WTO Panel Report 1998, para. 4.43; CJEU 1998, para. 97).

4 For example, the Confucian tradition links precaution with prudence/practical wisdom/Zhongyong and sincerity (Lai 2015, 75).

5 The European Commission’s Communication acknowledged that “the Community and the Member States have demonstrated the importance they attach to access to information and *justice*” (European Commission 2000, 16) [*Emphasis added*].

6 Some people premise the precautionary principle and its operation on “distributive justice” (Gardiner 2006; Ambrus 2012). Notwithstanding whether they are right or not which shall not be addressed here, naturally, although “justice”, “law”, and “governance” are different concepts, it is hard to believe that law and governance should be structure to injustice as opposed to justice (Rawls 2009; Fuller 1958, 636–37; Dworkin 2004, 24–25).

7 Sunstein acknowledged that the weak version is something “*no reasonable person could object*” (Sunstein 2003, 1012). Rejecting the precautionary principle *blanketly*, based on criticisms against *only* “the strong versions” is doubtful (Hughes 2006). Meanwhile, it is also strange to criticize a principle for various formulations. Each person, body of authority, or jurisdiction has the autonomy to articulate their own understandings, impacted by their lexicology resources and writing style. In fact, it is not rare for a phenomenon or principle to receive more than one understanding or description across jurisdictions and cultures, which should not and does not negate or invalidate the principle. It is also not common for every single person to accept only one universal precise formula of principles (otherwise, studies concerning conceptual matters or principles should not be demanded).
